# Domain‐topic models with chained dimensions: Charting an emergent domain of a major oncology conference

**DOI:** 10.1002/asi.24606

**Published:** 2021-11-24

**Authors:** Alexandre Hannud Abdo, Jean‐Philippe Cointet, Pascale Bourret, Alberto Cambrosio

**Affiliations:** ^1^ LISIS, Université Gustave Eiffel, INRAE Marne‐la‐Vallée France; ^2^ Garoa Hacker Clube São Paulo Brazil; ^3^ Sciences Po, médialab Paris France; ^4^ Aix Marseille Univ, Inserm, IRD, SESSTIM, ISSPAM Marseille France; ^5^ Department of Social Studies of Medicine McGill University Montreal Quebec Canada

## Abstract

This paper presents a contribution to the study of bibliographic corpora through science mapping. From a graph representation of documents and their textual dimension, stochastic block models can provide a simultaneous clustering of documents and words that we call a domain‐topic model. Previous work investigated the resulting topics, or word clusters, while ours focuses on the study of the document clusters we call domains. To enable the description and interactive navigation of domains, we introduce measures and interfaces that consider the structure of the model to relate both types of clusters. We then present a procedure that extends the block model to cluster metadata attributes of documents, which we call a domain‐chained model, noting that our measures and interfaces transpose to metadata clusters. We provide an example application to a corpus relevant to current science, technology and society (STS) research and an interesting case for our approach: the abstracts presented between 1995 and 2017 at the American Society of Clinical Oncology Annual Meeting, the major oncology research conference. Through a sequence of domain‐topic and domain‐chained models, we identify and describe a group of domains that have notably grown through the last decades and which we relate to the establishment of “oncopolicy” as a major concern in oncology.

## 1 INTRODUCTION

Building on the tradition of co‐word analysis (Callon et al., 1983), which can be considered the first attempt at using the content of documents to capture the dynamics of technoscientific activities, a variety of methods have been developed to reveal meaningful relationships between words, documents, and other dimensions of textual corpora, chiefly among them semantic maps and topic models (Blei & Lafferty, 2007; Leydesdorff & Welbers, 2011). Recent work (Gerlach et al., 2018) has shown that a family of network models, called Bayesian stochastic block models (Peixoto, 2018), offers an interesting topic modeling alternative to established latent dirichlet allocation (LDA) models (Blei et al., 2003). In this paper, we explore the fact that these network models can be employed to simultaneously infer document clusters, and we introduce procedures and tools to systematically interpret these clusters in combination with the topic model. We also introduce a method to extend this approach to other dimensions, thus covering a range of applications such as period detection and author topic models (Rosen‐Zvi et al., 2004).

The approach presented here represents an attempt to avoid the compromises of lacking a comprehensive statistical formulation, as with semantic maps, or treating documents as elements of a flat landscape, as in topic models. While topic models group words according to their patterns of occurrence in documents, our approach also accounts for patterns in documents' usage of words, gathering documents into groups we call the domains of a domain‐topic model. Other procedures that combine document clustering and topic modeling have been proposed, but based on stacking variants of LDA with models that produce document clusters, ranging from the naive application of k‐means on top of a topic model to more thoughtful, composed models such as MGCTM (Xie & Xing, 2013). Our goal in this paper is not to quantitatively compare our results to those procedures, but to explore the original affordances to augment and empower qualitative investigations that follow from the simplicity and flexibility of the present approach. Among such affordances, we show that the resulting lexically structured document landscape can be used as a lens to cluster and read other dimensions that converge in a document, such as its metadata, through what we will call domain‐chained models. And we translate our models into tables and interactive maps that visually tie clusters of documents with their lexical and metadata dimensions, allowing for the navigation and description of the multiple aspects of a corpus, at different scales.

To illustrate our approach, we selected a concrete research object that will be of interest to sociologists of the biomedical sciences. We investigate the abstracts of the annual meetings of the American Society of Clinical Oncology (ASCO) from 1995 to 2017 and show how we can lay out 23 years of the world's largest oncology conference in terms of research domains that rise, fall, or remain stable through a sequence of periods. We then proceed to study in detail one of the notable shifts, namely, the rise of “oncopolicy” as several associated domains, a central issue nowadays as ASCO officially transitions from being a “mostly research” to being a “research and policy debate” organization (ASCO, 2020).

It was during the preparation of this paper that Gerlach independently published (Gerlach et al., 2018) closely related work that covers the technique of applying the Stochastic Block Model (SBM) to a document‐term graph and demonstrates the validity of this approach as a topic model. Our work thus extends Gerlach's as outlined in this section.

## 2 METHODS

### 2.1 Domain‐topic models

We consider that research domains can be defined as sets of scientific texts addressing the same questions, using shared methods, and focusing on the same or related entities, all of which are reflected in the terms expressed in those texts. Our approach thus first relies on the content of documents to produce a simultaneous clustering of documents and terms, whereby documents are organized in domains that share a similar usage of topics and their terms, while terms are organized in topics that share a similar presence in domains and their documents.

**FIGURE 1:**
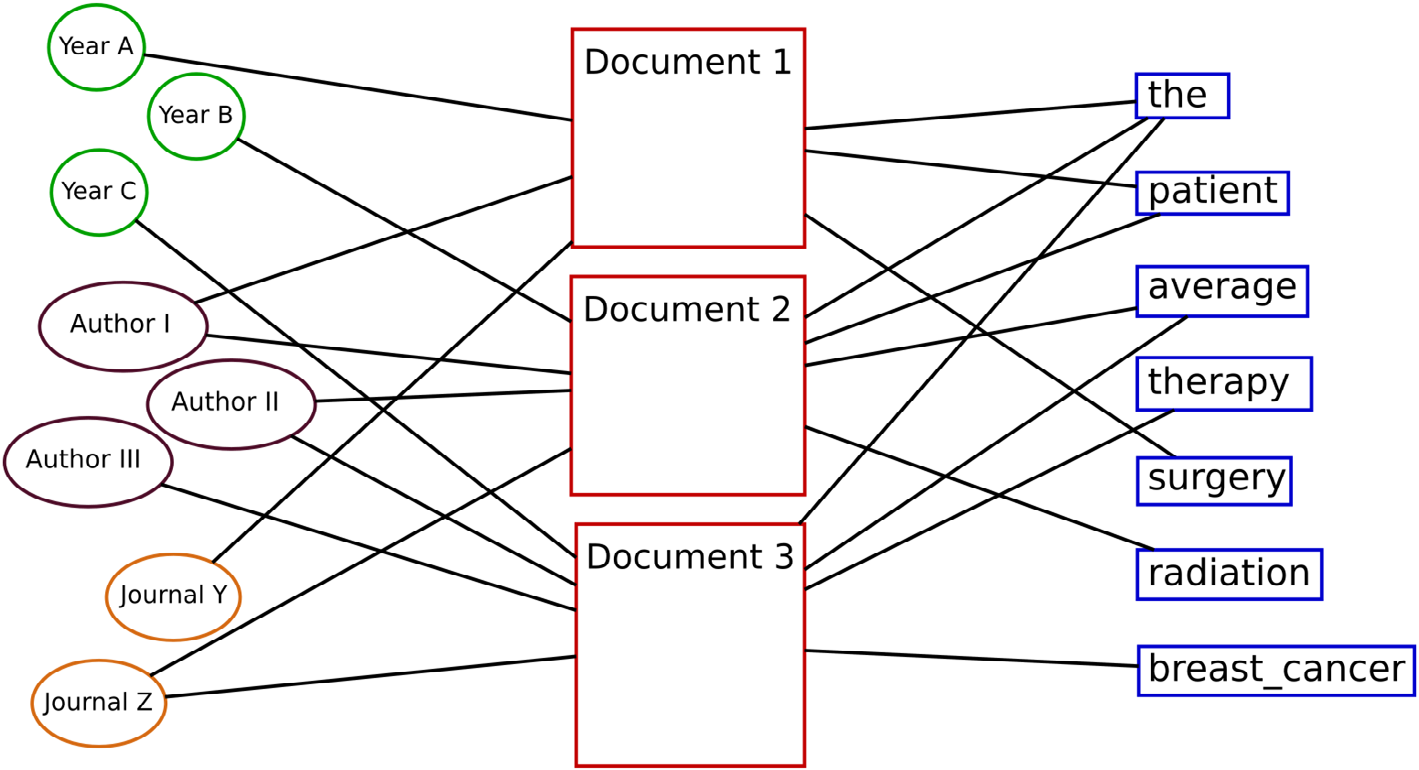
Incidence graph representation of relationships found in a research corpus. Each document appears as a node, with edges toward its textual content nodes (terms) and metadata nodes (e.g., authors, years, journals)

To analyze documents, we adopt a classic bag‐of‐words hypothesis (Harris, 1954) and model each document as an unordered set of terms, as exemplified in Figure 1, where for instance Document 1 is composed by the terms: “the,” “patient,” and “surgery.” In a graph representation, each document is thus connected to its terms, and also to its metadata dimensions, which in Figure 1 are: year, authors, and journal. This graph can be understood as the incidence graph of a hypergraph, whose nodes are the terms and metadata, and where documents are hyperedges associating multiple nodes. It is worth noting that an incidence graph is a bipartite graph: documents only connect to other types of nodes, and those only connect to documents.

**FIGURE 2:**
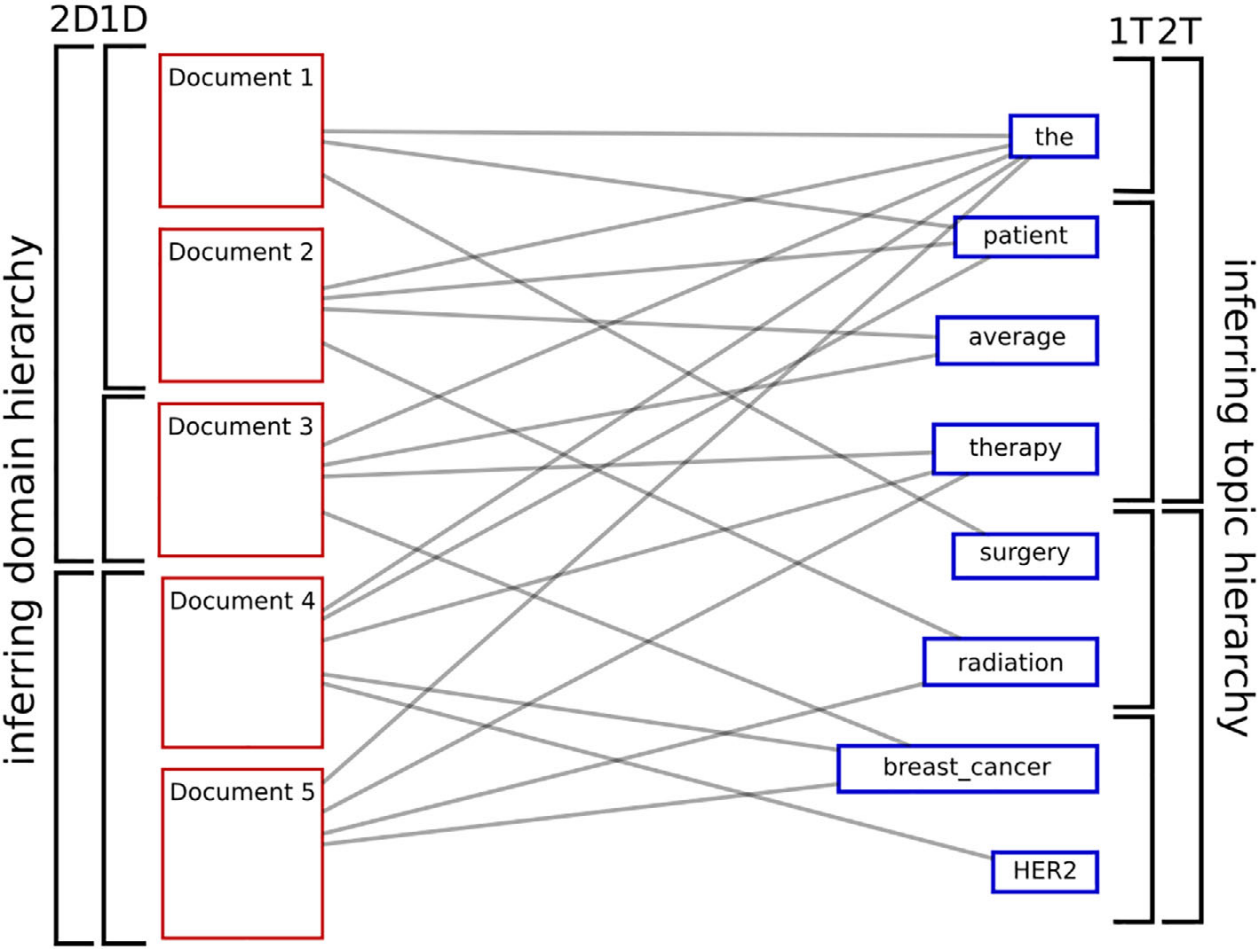
Domain‐topic model of the bipartite graph of documents linked to their terms. The resulting dual structure features on the right side a hierarchy of topics (groups of terms) and on the left side a hierarchy of domains (groups of documents). Labels 1D = “level 1 domains,” 2D = “level 2 domains,” and similarly for 1T and 2T as topics

In order to produce a domain‐topic model, we restrict this graph to its document and lexical dimensions, shown in Figure 2. We can then simultaneously cluster documents and terms to produce a categorization on both sides: documents are organized in domains, while terms are organized in topics, as depicted by the brackets in the figure. Note that Figure 2 portrays a nested organization, with two levels for domains and topics, where clusters get clustered themselves at higher levels, providing a description at multiple scales, as afforded by the clustering method discussed below.

One could, in principle, use any graph clustering method to deal with this task. However, the empirical difference in connectivity patterns on the two sides of this bipartite network raises the following issue. While terms may vary from being present in only a few documents to spanning all of them, the number of terms per document in a corpus will fluctuate around some average. Put differently, in a typical document‐term graph, the degree distribution of terms is wide and fat‐tailed, while the degree distribution of documents is narrow. This suggests that we will either need to couple two separate models or employ a model that can capture quite general patterns. Given that we also plan to treat different kinds of metadata as part of our approach, the latter option is clearly to be preferred.

In this paper, we adopt the Bayesian Stochastic Block Model (Peixoto, 2014b) as our graph clustering model, which, as required, clusters nodes according to general connectivity patterns, while also providing a parsimonious method for model fitting and selection. Specifically, we adopt its nested, degree‐corrected variant. In SBM terminology, node clusters are called *blocks*. The basic SBM is a generative model where nodes are organized into blocks and connected according to edge probabilities between those blocks. Degree‐correction improves on that by accounting for heterogeneous degree frequencies within blocks. The nested quality of the model means it expresses patterns at different scales, by clustering blocks themselves into higher‐level blocks, and so forth, forming a nested hierarchy. This stochastic model and related variants have been successfully applied to the analysis of both static and time‐varying graphs and have been shown to robustly reveal nontrivial connectivity patterns while avoiding overfitting (Peixoto, 2015b).

In this SBM framework, fitting the model to network data is done by partitioning (grouping) the graph's nodes into blocks. In the nested case, this includes further partitioning the blocks themselves into higher‐level blocks for each nested level. Given these partitions, their blocks' degree sequences and the connection probabilities between blocks are simply traced from the edges in the graph. A model that best fits the graph is then searched following a minimal description length (MDL) approach, by seeking a partition that minimizes the combined informational costs of describing the graph given the model, and of describing the choice of partitions and other model parameters (Peixoto, 2014b). Notably, both the number of levels in the hierarchy and the number of blocks at each level are inferred from the data through the MDL principle.

This class of models provides our domain‐topic model with a set of desirable properties:

- They detect *general connectivity patterns*; resolving the issue of treating different types of nodes: documents, terms, and metadata.
- They are *nonparametric*; in particular, both the number of blocks and levels in the hierarchy are directly inferred from the data.
- They do *not overfit*: by accounting for the information cost of model parameters when maximizing model probability, they only infer statistically significant structures.
- They provide a nested, multi‐level abstraction of terms and documents, allowing the investigation of topics and domains at different scales.

Another major concern in the study of corpora is the ease and reproducibility of result interpretation. This concern guides the following two additional choices regarding the model. First, stochastic models can be employed either by searching for a single model that best fits the data or by averaging the values of interest over a distribution of models yielded according to their fitness. In this paper, we choose to work with the single best‐known fit for our data. Second, the model adopted allows for overlapping as well as nonoverlapping blocks. In this paper, we have worked with nonoverlapping blocks. Contrary to what one might expect, it has been shown that nonoverlapping models are often a better fit than overlapping ones (Peixoto, 2015a). This remains an area for future work, including the development of models that overlap topics but not domains, to better take into account the polysemy of terms. Together, these two choices greatly simplify interpretability insofar as each document is attached to a single domain, both probabilistically (single best fit) and concretely (nonoverlapping).

In conclusion, we obtain our domain‐topic models by fitting the aforementioned choice of SBM to a document‐term graph, as pictured in Figure 2. We perform our optimization procedures using the SBM implementation found in the graph‐tool library (Peixoto, 2014a), which employs an efficient Markov Chain Monte Carlo approach. Our usage of the library and the specific procedures adopted for this paper are detailed in the Appendix S1 (in SI‐DATA [SI‐DATA, SI‐MAPS and SI‐TABLES available from https://doi.org/10.5281/zenodo.3596035]). The work presented here has received ethical approval by the Institutional Review Board of the Faculty of Medicine of McGill University (IRB Study Number A07‐E55‐15B).

### 2.2 Chained dimensions

Research domains also carry social and contextual dimensions, given that they are grounded in the historically situated, socially contingent activities of specific teams and institutions, whose results are made public at professional conferences and in scholarly journals. These dimensions are typically reflected in metadata such as authorship, institutional affiliations, funding sources, or year of publication. Our present purpose is to study them through the lens of the substantive content of the corpus, that is, we want to cluster metadata elements that entertain similar connections across the domains of a domain‐topic model.

**FIGURE 3:**
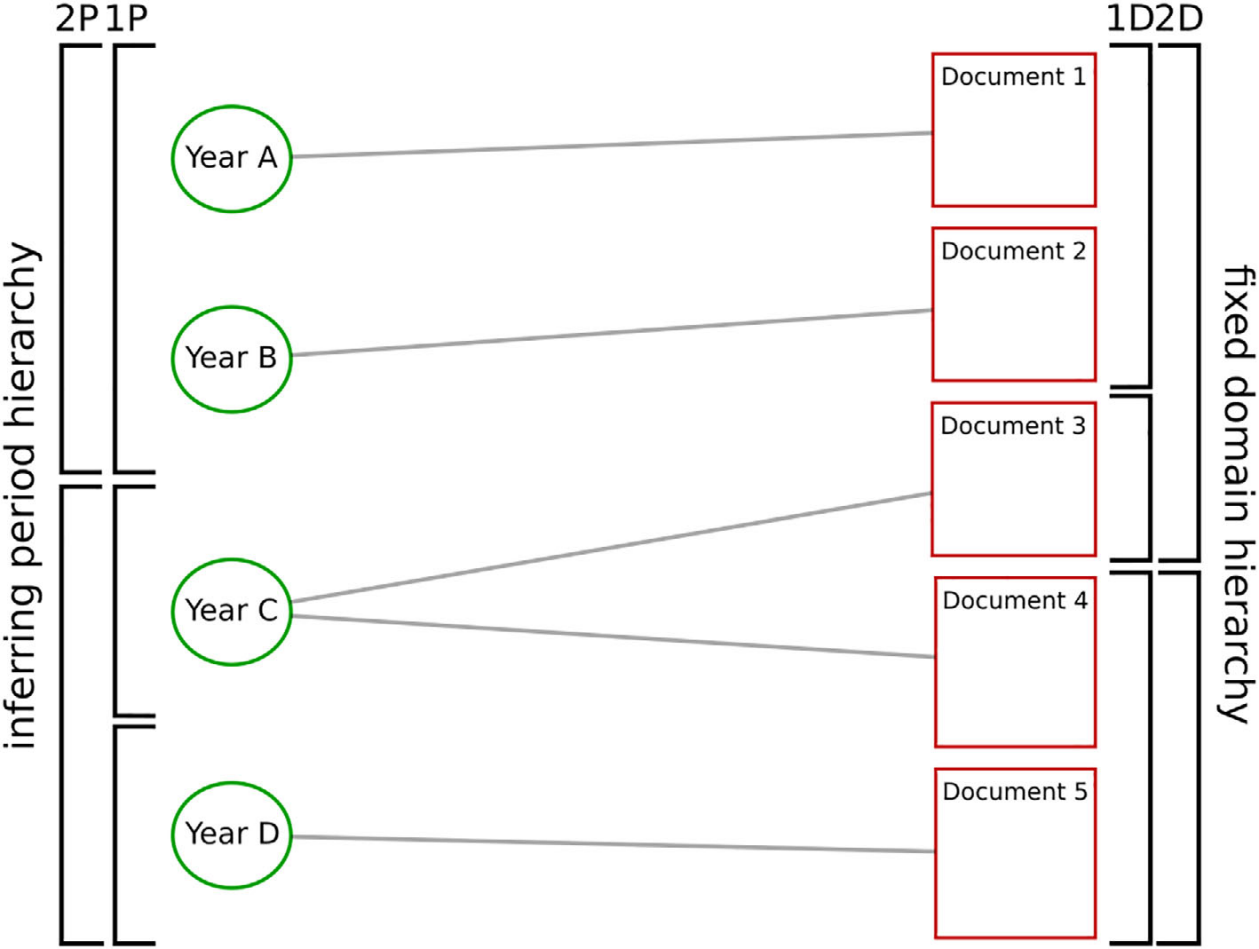
Domain‐chained model of documents linked to their year of publication. By keeping the inferred document partition fixed, the model can be extended to other variables that get assigned to documents. In this example, publication years get partitioned into nested blocks. The best fit partition will reflect the connectivity patterns between the chained dimension and the lexically structured domains (1P = level 1 periods, 2P = level 2 periods)

To achieve that, after having inferred a domain‐topic model for a given corpus, we use its domains to form an *inference chain* toward the documents' metadata. We begin by restricting the original graph (Figure 1) to a given metadata dimension, with documents connecting to their metadata nodes, as can be seen in Figure 3. We then fit the SBM to this new graph, but transposing and keeping immutable the nested blocks of documents previously inferred by the domain‐topic model. Consequently, only the metadata nodes get partitioned, as represented by the brackets on the left side of Figure 3, and the best fit partition will reflect their connectivity patterns to documents and the domain hierarchy. We call this procedure a domain‐chained model.

### 2.3 Domain tables and interactive maps

A good model of a process is not necessarily useful if it does not come with the appropriate tools to make sense of the resulting representation. For our inferred domains, topics, and metadata blocks, we must provide interfaces that effectively deploy these abstractions as lenses through which one can interact with and interpret the data, in conjunction with the relationships between different block types at multiple levels.

Documents are the main object of interpretative interest in our approach. They are the material product assembling terms into meaningful texts and connecting the many dimensions of a corpus. It is thus the interpretation of domains, as sets of documents manifesting assemblages of topics characteristic of the corpus, that will allow us to dissect the data and make sense of their evolution and their nontextual dimensions. In this section, we present two related interfaces for interpreting domains: domain‐topic tables and domain‐topic maps.

The ability to discuss blocks at different scales, as provided by the nested aspect of the model, plays a key role in these maps and tables. We will talk of a superdomain and a subdomain to refer to, respectively, the parent and one child of a domain in the nested hierarchy. Level 1 domains have no subdomains, as they directly partition documents (see 1D in Figure 2). Conversely, the single domain at the highest level has no superdomain, since it contains all documents. The same goes for subtopics and supertopics and, more generally, for superblocks and subblocks of any dimension.

While working at different scales, one must remain attentive to the privileged role of level 1 blocks. In the generative process of the nested model, concrete document‐term links are added exclusively from probabilities of connections between level 1 blocks. At higher levels, blocks gather in superblocks whose connections are the combined connections of their children, but such combined connectivity pattern has no requirement to represent concrete nodes in the data. To illustrate this issue, consider that a level 2 domain may have two subdomains D1 and D2, where topic T1 is absent from D1 and topic T2 is absent from D2. The topic distribution of the superdomain, alone, would have us falsely believe that T1 and T2 are employed together. Therefore, the meaning of higher‐level blocks must be built up from their base nodes. It is at the level 1 domain that we find, in the content of its documents, a concrete and coherent assemblage of topics. Likewise, it is within level 1 topics that we find terms with a coherent tendency to be employed in some domains but not in others.

As a consequence, for an interface to properly assist in the interpretation of blocks, it must tell us which level 1 topics are distinctive of a given level 1 domain, as we can expect such topics to be articulated together within the domain's documents. For higher‐level domains, however, it is more meaningful to ask what their constituent parts have in common, that is, what topics are distinctive of all of their subdomains. These observations translate into two information theoretical measures that we will employ in our interfaces: the nested specificity and nested commonality. We shall first formally define these measures, before following with a presentation of the domain‐topic tables and maps.

#### 2.3.1 Nested specificity and nested commonality

To express specificity, let us consider a domain and its nested superdomains as probability distributions over level 1 topics, taken from the frequency of usage of those topics in their corresponding documents. We can then resort to the relative entropy (the Kullback–Leibler divergence) between these distributions, in order to quantify the contribution of each topic to the overall information gain incurred when we describe topic probabilities using the domain's actual distribution instead of a higher‐level superdomain's broader distribution. That contribution is the expectation‐weighted pointwise relative entropy of a topic. For the nested specificity, to account for all scales while still accentuating more local ones, we average this contribution across the superdomain ladder. The nested specificity of a topic t for a domain d, with $d_{+}⊃d$ representing the domains $d_{+}$ that are superdomains of d at each level above it, can thus be expressed as:

$${\hat{S}}_{d}\left(t\right)=\frac{1}{|d_{+}⊃d|}\sum_{d_{+}⊃d}p_{d}\left(t\right)\log\left(\frac{p_{d}\left(t\right)}{p_{d_{+}}\left(t\right)}\right).$$

For the nested commonality, we want to express the extent to which a topic has high specificity for all subdomains of a domain, in respect to its superdomains. To that end, we define a quantity related to the change, when replacing distributions by that of a superdomain, in the probability of always sampling the topic in question if one samples one topic from each subdomain. That quantity is the (unweighted) pointwise relative entropy of a topic between a subdomain and a superdomain of the domain, averaged across the subdomains, which we then averaged across the domain's superdomain ladder. At a topic t for a domain d, with $d_{+}⊃d$ as before and $d_{-}\subset d$ standing for the subdomains $d_{-}$ of d at the level immediately below it, we have:

$${{\hat{C}}^{*}}_{d}\left(t\right)=\frac{1}{|d_{+}⊃d|}\sum_{d_{+}⊃d}\frac{1}{|d_{-}\subset d|}\sum_{d_{-}\subset d}\log\left(\frac{p_{d_{-}}\left(t\right)}{p_{d_{+}}\left(t\right)}\right).$$

It is positive if a topic is overrepresented in all subdomains, and negative if sufficiently underrepresented in at least one subdomain, reaching minus infinity if the topic is missing from any of them. Note that we average over the domain's superdomain ladder, not including the domain itself, to not accentuate the specificity of subdomains toward their own union, which would run contrary to our goal of highlighting their shared specific topics.

We must now account for expectation, so that our nested commonality measure corresponds to a topic's contribution to this quantity's expected value. Consistent with the above, we treat the subdomains as equivalent units, and consider a topic's probability as the probability of first uniformly choosing a subdomain, and then sampling a topic from it. We finally obtain:

$${\hat{C}}_{d}\left(t\right)=\left(\frac{1}{|d_{-}\subset d|}\sum_{d_{-}\subset d}p_{d_{-}}\left(t\right)\right){{\hat{C}}^{*}}_{d}\left(t\right).$$

While we have developed these measures to obtain characteristic topics for domains, the symmetric structure of the nested blocks actually affords repurposing them for any two block types, such as to obtain characteristic domains for metadata blocks, and even characteristic domains for topics. It is also straightforward to replace a cluster type with its elements, for example, for characteristic terms instead of topics.

#### 2.3.2 Domain‐topic table

The domain‐topic table answers the need for a simple, static, and publication friendly interface. It features a chosen group of domains together with their constitutive level 1 subdomains, and it uses the measures defined above to list, for each domain in the group, the topics common to its immediate subdomains, as well as the specific topics of the level 1 subdomains. For example, if we are interested in a level 3 domain, the domain‐topic table lets us examine the group of its level 2 subdomains. For each level 2 domain, it would display what its subdomains have in common, and what is specific to the level 1 domains composing it. In this case, the table can also present what the level 2 domains have in common among themselves, as subdomains of the level 3 domain (Table 1). Schematically, we have:

**TABLE 1:** Domain‐topic table for the level 2 subdomains of a level 3 domain, showing common topics for domains at levels above 1, and specific topics for level 1 domains

[Level 3 domain]			
[Common topics of its level 2 subdomains]			
[Level 2 domain]	[Common topics]	[Level 1 domain]	[Specific topics]
		[Level 1 domain]	[Specific topics]
[Level 2 domain]	[Common topics]	[Level 1 domain]	[Specific topics]
		[Level 1 domain]	[Specific topics]

In such a table, domains are represented by their index, while topics are represented by their index and a list of terms. We use the following criteria to choose which topics and terms to display: for topics, we draw from the topics with highest specificity (or commonality) for the domain in question, until those topics account for half the sum of the positive specificities (or commonalities) over all topics. For terms from a topic, we pick those terms whose values are higher than half the highest value. The difference in criteria for topics and terms are a consequence of what they represent: distinct topics display different connectivity patterns, so we account for enough topics to cover a majority of their weight; meanwhile, terms within the same topic display similar connectivity patterns, so we simply account for those that contribute the most.

As with our two measures, these tables can be similarly constructed for other pairs of block types.

#### 2.3.3 Domain‐topic map

**FIGURE 4:**
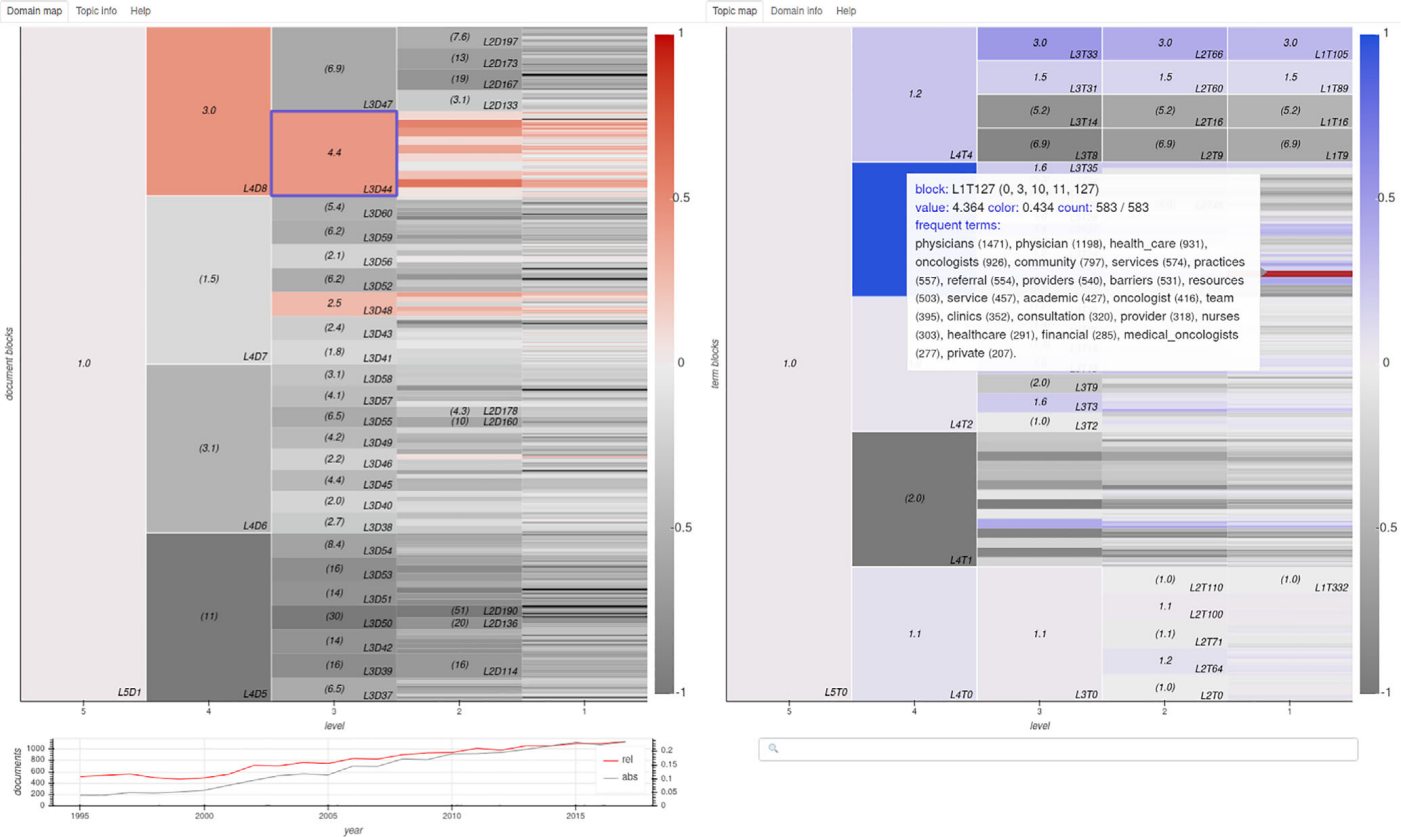
Screen capture of a domain‐topic map, with longitudinal histogram and term search. Domains are shown on the left, in red, and topics on the right, in blue. Columns show the partitions at decreasing levels of the nested hierarchy, where each block is sliced into subblocks with equal heights. In the figure, domains appear colored for their usage of the selected level 1 topic, associated with health care, and topics appear colored for their usage in the selected domain, which happens to be the level 3 domain most associated with the selected topic

Domain‐topic tables are useful when one already has a broad understanding of the corpus they are studying, and has chosen a set of domains to investigate. But we still need a tool for interactively exploring the corpus in its different scales and dimensions. Domain‐topic maps provide such a tool, depicted in Figure 4.

In them, color represents the relevance of a block, normalized so that the greatest value within each level corresponds to the strongest color. Before selecting a block, this relevance corresponds, for domains, to the fraction of the corpus they contain, and for topics, to the fraction of term usage originating from that topic. By interacting with the map, one may:

- cursor over a domain: displays the domain's specific (or common) topics and terms.
- cursor over a topic: displays the most frequently employed terms from the topic;
- scroll: zooms in and out of lower levels, following the hierarchy;
- select a block:
  - changes the opposite map to display colors relative to the block, see Figure 4;
  - in a secondary view, displays general information on the block plus titles of pertinent documents, linked to their URL;
  - when selecting a domain, restricts the histogram to its documents.
- search: selects a topic by entering a term it contains.

These maps can also be built pairing domains and metadata blocks, providing the same kind of interactivity.

## 3 DATA

### 3.1 The ASCO annual meeting abstracts

To test and exemplify our approach, we analyzed a dataset of conference abstracts from the ASCO Annual Meeting between 1995 and 2017.

Why conference abstracts rather than journal articles? First, because scientific and clinical gatherings are a major forum for the introduction of the latest clinical and scientific research results, including some that are preliminary, will not necessarily be confirmed, and will therefore remain unpublished (Massey et al., 2016). Investigating conference abstracts rather than publications thus provides a privileged take on “science in the making” (Latour, 1987), that is, in our case, the moving front of oncology research, while also opening the possibility of comparing different stages of the production of scientific knowledge. Second, to explore and highlight the fact that content‐based methods require only the text of individual documents, rather than the presence, machine‐readability and uniformity of specific metadata such as citations or co‐authorship. Our approach is thus applicable to many kinds of documents, including publications, but also grant proposals, historical archives, and particularly, to conference proceedings.

**FIGURE 5:**
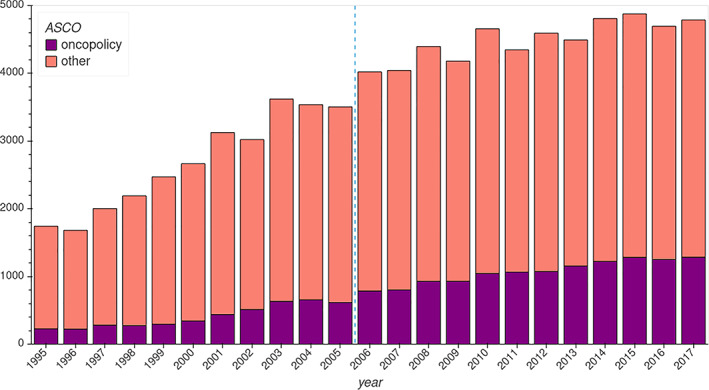
Abstracts presented at the American Society of Clinical Oncology Annual Meeting between 1995 and 2017, totaling 83,476. Advancing some of our results, we highlight the contribution of a group of domains we label “oncopolicy” and show the split between the two main periods detected

And why ASCO? In the Appendix A1, we provide a field knowledge backed account of the importance of the ASCO Annual Meeting for oncology research and translational science, and of why its abstracts are both a relevant and an appropriate corpus to understand the field's global research front. Figure 5 shows the evolution of the number of abstracts presented at the meeting from 1995 to 2017, reaching a plateau of approximately 5,000 abstracts in the 2010s, while in 2018, the number of conference participants surpassed 40,000, with half of them international.

Because our corpus consists of abstracts, and being the only form in which these conferences are archived, we acknowledge the broader issue of whether abstracts can be considered representative of the content of full conference presentations or, for that matter, of published articles. Recent evidence (Ermakova et al., 2018), albeit based on journal abstracts from a different field, shows that abstracts cannot be considered as “mere teasers,” and that in fact their “generosity,” defined on the basis of the amount and importance of information provided by the abstracts, has been increasing in recent years.

### 3.2 Data processing

This section describes our processing of the ASCO abstracts in preparation to infer their domain‐topic block structure. To further demonstrate the flexibility afforded by our adoption of a statistical model capable of detecting generic connectivity patterns, we opt for a minimalistic preprocessing strategy that is context and language independent. This is not to say that contextual preprocessing would not be helpful, but rather to stress that it is not as necessary. We therefore do not filter language or corpus‐specific stop‐words and low frequency words, and instead we let the model identify these patterns, which it successfully separates from more relevant terms by assigning them their own topics. Using the notation introduced in the following section, those topics are, respectively, L1T332 and L1T0. By contrast, in the case of LDA topic models this would require context‐specific preprocessing or modeling choices (Wallach et al., 2009). We also do not apply language‐specific natural language processing (NLP) text transformations such as stemming or lemmatizing.

Other than tokenization of the text, our preprocessing thus consists of two steps. First, we search for co‐location using classical statistical analysis (Mikolov et al., 2013) to identify and replace frequent bigrams in the corpus such as “stem_cells” and “breast_cancer.” While we could have identified higher order *n*‐grams, we consider that bigrams are both common practice and sufficient, as the benefit of employing higher order *n*‐grams overlaps with that of the term clustering performed by the model. Second, we build for each abstract a list of its terms, discarding repetitions. By ignoring the local frequency of terms, we put more weight on the thematic features of the text and avoid patterns based on stylistic variations like preferences to replace nouns by pronouns. We do not argue that these are the best choices, only that they make sense in our case of quite uniform documents and for the purpose of illustrating what can be achieved with minimal dependency on additional procedures.

## 4 RESULTS

We adopt the following notation to refer to individual blocks at a particular level:

LiDj≡domainjatleveli.

LiTj≡topicjatleveli.

For instance, L2T29 is the topic with index 29 at level 2, and L3D40 is the domain with index 40 at level 3. Blocks are indexed in such a way that, for a given level, domain indices start from the highest topic index.

**FIGURE 6:**
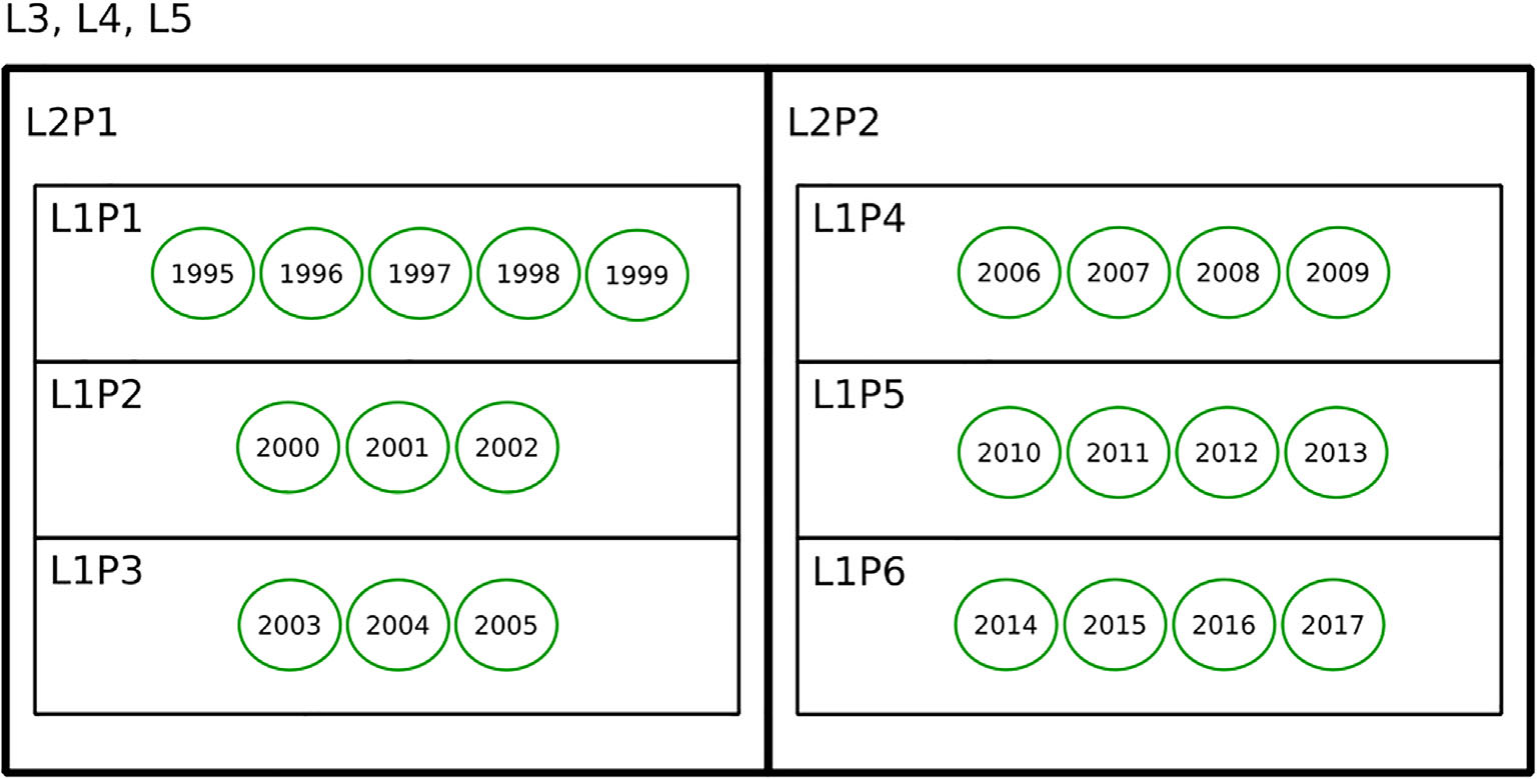
The nested partition of conference years into periods. Contrary to domains and topics, levels L3, L4, and L5 are all equivalent, as the inference procedure found no statistically significant distinctions above level L2. Moreover, since years are treated as categorical data, the fact that the partitions respect the chronological sequence is not a given, but reveals a progressive character in the evolution of research domains at the American Society of Clinical Oncology Annual Meeting

To begin our analysis of the ASCO Annual Meeting abstracts, we produced a domain‐topic model of its contents, of which Table 2 shows the block counts, and then produced a domain‐topic map to work with, which readers can access in Appendix S1 SI‐MAPS to gain a first‐hand experience. Additionally, we produced a domain‐chained model of the meeting year associated with each abstract, then produced a domain‐period map, also available in Appendix S1 SI‐MAPS. We depict its nested blocks, which we call periods, in Figure 6.

**TABLE 2:** The base number (*N*) of documents and terms, followed by the number of partitions in domains and topics at each nested level of the domain‐topic model

	*N*		L1	L2	L3	L4	L5
Documents	83,476	Domains	479	110	24	4	1
Terms	253,758	Topics	407	112	37	5	1

**FIGURE 7:**
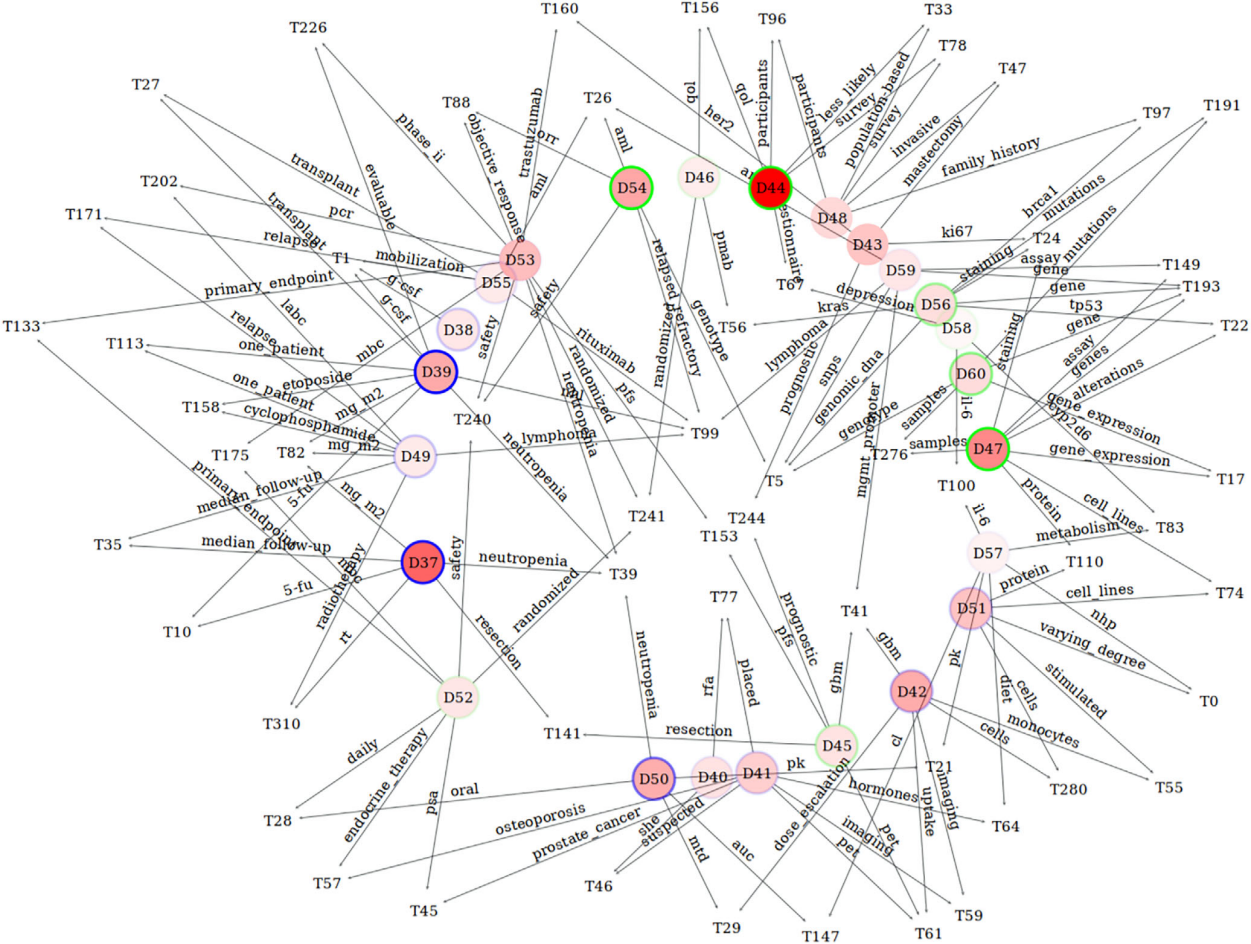
Domain‐topic network showing connections between level 3 domains and their shared most specific level 1 topics. For each domain node, the strength of the red filling corresponds to the volume of documents belonging to the domain, and the border color represents the intensity of its growth (green) or decline (blue) between periods (1995–2005) and (2006–2017). The label on each edge shows the word from the topic most specific to the domain it connects. As an example, at the bottom we see D50, which has an intermediate total volume and strongly decreases between the periods. T29 is a specific topic for D50, with “mtd” (for “maximum tolerated dose”) its most specific term for this domain

Before introducing a more rigorous and interactive procedure for inquiry, in the interest of providing a simple overview of the corpus, we constructed a network image of the connections between level 3 domains and their most specific level 1 topics, presented in Figure 7. The choice of level 3 for domains is imposed by the number of domains that can be presented in an image, while the choice of level 1 for topics is imposed by the fact that, as previously discussed, topics of this level form concrete and interpretable sets of terms. To further render the image readable, we also removed topics that were not shared between at least two domains.

To the left of the image, we see an ensemble of voluminous yet strongly decreasing domains, whose topics are suggestive of clinical trials of traditional chemotherapy and radiotherapy treatments concerned with dosages, neutropenia, etc., from the largest and strongly decreasing D37 up to the rather stable D53, which already links to modern targeted therapies. This diminishing sector extends to the bottom of the image, where we find an ensemble with more specific concerns, such as “mtd” (maximum tolerated dose) and “pk” (pharmacokinetics) for D50, “rfa” (radiofrequency ablation) for D40, and imaging, hormones, and hormone‐related cancers (e.g., prostate cancer) for D41. In between those sectors, we see the small but increasing D52, linked to trials of more recent hormonal treatments for prostate cancers, and to their right also increasing is D45, associated with modern imagery and surgery for brain cancers. Still toward the lower right, a small group concerns the study of cells and their physiology, from D42 to D57, showing a lesser decrease. This latter group shares aspects with the large and strongly rising D47, associating cells, and genetics. From D47 to D43, we can see on the upper‐right the rising world of genetics and genomics, complemented by the relatively large D54, which ties clinical trials to genotyping, besides which we find and small domain, D46, linked to “qol” (quality of life) and clinical trials. Finally, toward the top we find two domains, D44 and D48, sharing concerns about populations and participants, but with a different emphasis (see below our discussion of “oncopolicy”).

We start our inquiry by employing the domain‐topic and domain‐period maps. Because domain‐topic maps offer the opportunity to delve into the details of the entire corpus, they afford two complementary disciplined uses: on the one hand, they allow researchers to perform an exhaustive analysis of its domains, which in our case encompasses the numerous developments at clinical oncology's research front over 23 years; and, on the other hand, they allow researchers interested in a specific set of questions to uncover the relevant domains on which to focus their analysis. The present paper explores this latter usage, because it allows us to demonstrate the specific task of determining what portion of the map to focus on, but also to exemplify the tasks and procedures involved in the exhaustive approach while conveniently restricting our scope to fewer domains.

**FIGURE 8:**
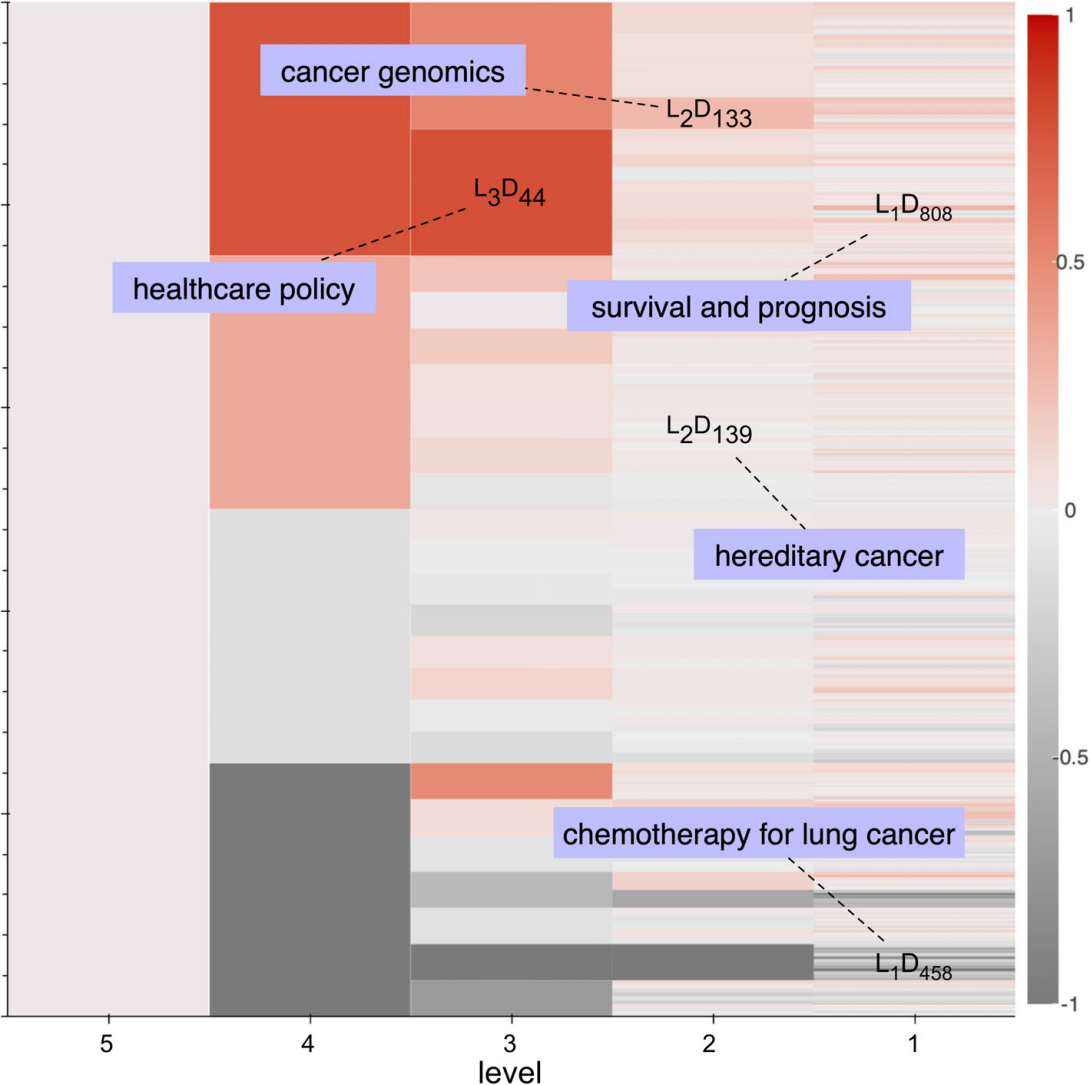
Colors represent the growth, in red, or decline, in gray, of the prevalence of domains between periods (1995–2005) and (2006–2017). Labels are the result of procedures akin to what we will perform for L3D44

We proceed by navigating the domain‐period map in order to examine which research domains experience meaningful shifts in their prevalence between the different periods. We can formalize this notion by measuring the difference of a domain's prevalence between subsequent periods. In this way, we obtain the color map in Figure 8, showing the growth or decline of domains between the major periods, (1995–2005)^L2P1^ and (2006–2017)^L2P2^. We have added labels singling out some notable domains. At level 1, the domain with the highest growth refers to survival and prognosis across different cancers^L1D808^, a finding that corresponds to a renewed interest in prediction as discussed in (Christakis, 1999). Still at level 1, the domain with the strongest decline refers to traditional chemotherapy for lung cancer^L1D458^ and is consistent with the demise of cytotoxic chemotherapy approaches at the research front. At level 2, the highest growth corresponds to cancer genomics as defined by work on genomic alterations, mutations, and molecular profiles across cancer types^L2D133^, a finding consistent with the meteoric rise of precision oncology. We also find a very stable domain that corresponds to the long‐lasting interest in hereditary cancer^L2D139^.

Although trends such as the ones we just mentioned—an increased focus on prognosis and prediction, the decline of traditional lung cancer chemotherapy (replaced by targeted drugs and immunotherapies), or the meteoric growth of cancer genomics—are far from surprising for readers familiar with oncology, the fact that the domain‐topic model was able to capture them demonstrates its ability to single out key developments that have characterized the field. Besides these well‐known trends, we also find surprising or less obvious ones. In particular, at level 3 we notice a high‐growth domain, L3D44, that we initially had trouble characterizing, as it consists of a number of miscellaneous contributions related to public health, healthcare policy, healthcare services, and cost analysis. Not only did this domain refer to issues that one would not necessarily expect to see discussed at a clinical research meeting, but, as we will see below, it also appeared to do so by embedding these policy topics into activities such as clinical trials or other biomedical investigations such as epidemiological surveys. This particular result prompted us to launch a qualitative investigation that led us to a set of activities to which oncologists refer by the umbrella term “oncopolicy.” It is important to emphasize at this stage that the qualitative investigation was spurred by the computational results and, in turn, as detailed below, led us back to a computational exploration, resulting in an iterative process of aligning these two approaches (Leydesdorff et al., 2020).

Pursuing our qualitative remarks, the term “oncopolicy,” which entered common biomedical parlance around 2013, refers to a somewhat amorphous field, a liminal space where oncologists interact with policymakers and other stakeholders in order to shape matters related to health and research. The common goal is to contribute proactively to the development of oncology's care and research priorities while by the same token turning this multidisciplinary specialty into a recognized component of health and research policy agendas. Beyond the generic goal of “bridg[ing] the gap between science and policy” (ECCO, 2012), oncopolicy is designed to address a number of different issues ranging from personalized medicine and care to the organization of specialized care, and the translation of evidence into policy. Around 2012, following discussions about “being bolder with policy issues,”, ASCO's Board established a new position of Chief Medical Officer to focus on “quality programs, public policy, communications, and fundraising” (Rosenthal, 2012), thus providing “oncopolicy” (albeit without using the neologism) with an institutional bedrock, a move confirmed by the establishment in 2017 of a health policy fellowship and, more notably, the launch in 2020 of a twin “professional association [to] enable expanded advocacy activities and increase the impact of efforts directed toward policymakers in support of high‐quality patient care” (ASCO, 2020). In short, the growing domain we encountered corresponds to a major reorientation currently taking place not only at ASCO, but also within other oncology organizations such as the National Comprehensive Cancer Network (McNeil, 2018). As a result, health policy topics that a few years ago would not have been discussed at meetings have become legitimate topics as part of the present “era of rapid change—in therapies, costs, payment models, and practice.” To conclude this account, we note that our computational analysis brought the “oncopolicy” issue to our attention based on data from a period that predates the above major developments of recent years.

So far, we have employed the domain‐topic and domain‐period maps to get a broad understanding of domains that underwent notable shifts in prevalence between the two main periods. Through that, we encountered an unexpected domain, and qualitative inquiry provided us with an initial understanding of its significance and contours, confronting us with an issue worthy of attention. It is toward this domain and the issue it raises that we now turn for an in‐depth analysis of the inferred domains and topics.

The domain L3D44 contains 10 level 2 domains, each with its set of level 1 subdomains. Our starting goal is to provide, for each level 2 domain, a label that accounts for the abstract coherence between its level 1 subdomains. This will not only analytically describe L3D44 in terms of 10 meaningful domains, but also allow us to answer how those domains are related to other dimensions. To produce these labels, we resort to the domain‐topic table for domain L3D44. We refer readers interested in following the analysis of domain‐topic tables to produce domain labels to Appendix B1, where we also present the procedure and rationale to identify and include in our analysis a second related level 3 domain, L3D48.

**FIGURE 9:**
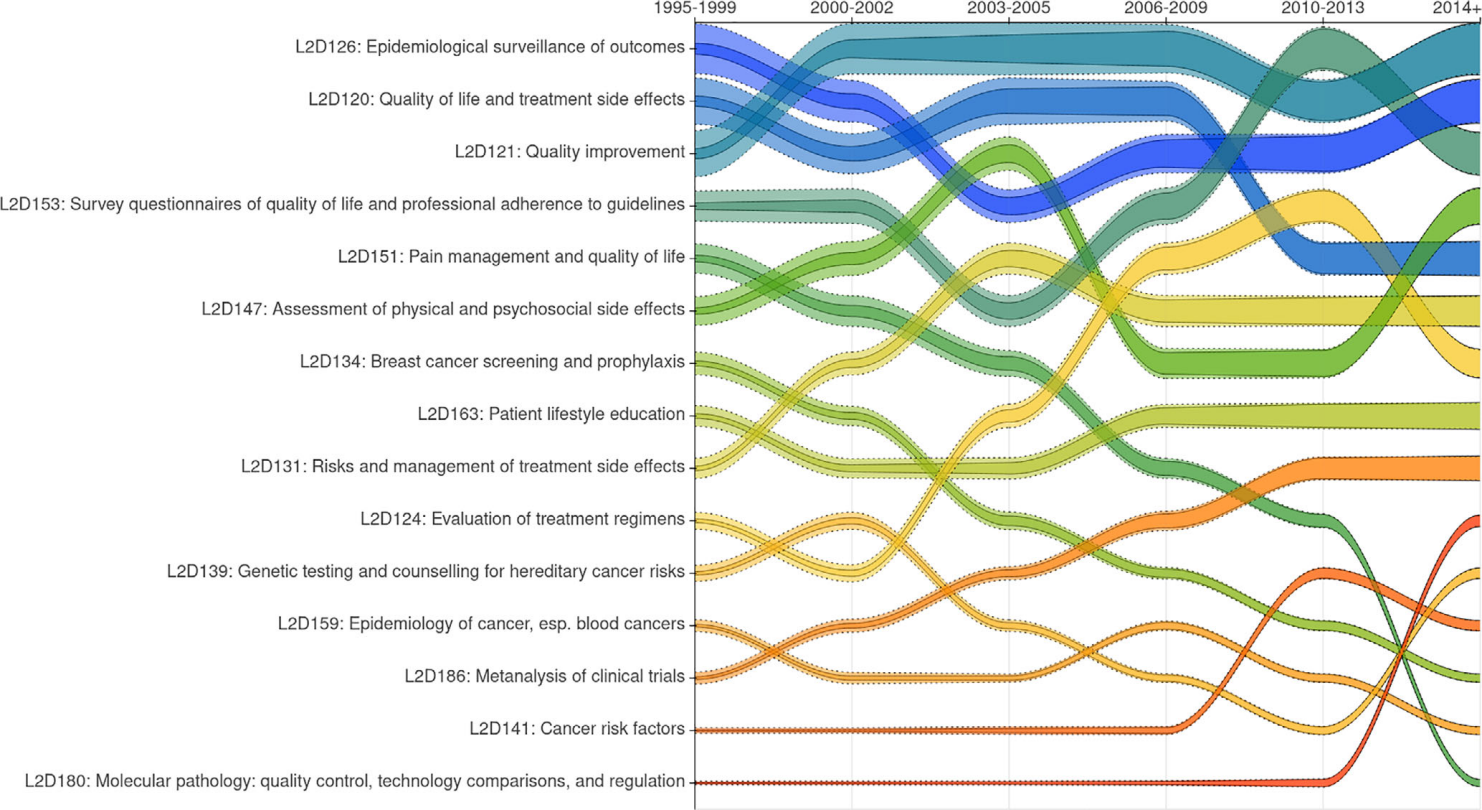
Area bump chart for the 15 level 2 domains of “oncopolicy” (L3D44 and L3D48), displaying rank and volume changes along the 6 level 1 periods. Volumes are year averages within each period, and both absolute (continuous line) and relative (dotted line) volumes are shown for each domain

At this point, we examined the oncopolicy arena as defined by the combination of two level 3 domains: “public health and health technology assessment”^L3D44^ and “screening and risk factors for cancers”^L3D48^. We carefully labeled their subdomains to elicit a set of meaningful and related, yet distinctive aspects, and we can now proceed to the description of its emergence, evolution, and transformation. To do so, we resort to an area bump chart (Figure 9) of the 15 level 2 subdomains across the 6 previously inferred level 1 periods (see Figure 6), that is, a plot of lines, one for each subdomain, whose thickness reflects their absolute volume (solid border) and relative volume (dotted border) at each period. The lines are ordered for each period from highest to lowest volume, so that each subdomain's changes in rank over time are indicated by line crossings.

As indicated by the top portion of the bump chart, issues related to quality of life^L2D120,L2D153^ and the quality of professional practices and treatments^L2D121,L2D153^, together with the epidemiological surveillance of treatment outcomes^L2D126^, occupy a strong position within the oncopoplicy arena both in terms of rank and volume. At the same time, even as oncopolicy developed into a major subject within ASCO, the overall distribution of its subdomains' proportions has been remarkably stable, revealing that its rise cannot be attributed to some fast‐growing subset of these domains, but rather suggesting that its diverse aspects evolved together. In other words, we can hypothesize that oncopolicy amounts to a slow‐accretion assemblage that originated in an early concern for improving the quality of patient care and, by extension, the quality of life of patients. The inclusion of other subdomains related to this same fundamental concern shapes the assemblage in relation to the transformation of the technoscientific arsenal of oncology, but also to the multiplication of its stakeholders and, in particular, to a more vocal presence of the patients. Looking at the subdomains that have most recently increased their rank, we notice molecular pathology^L2D180^ with its focus on the regulation, broadly speaking, of the fast‐growing field of molecular testing, and the cancer risk‐factors subdomain^L2D141^ as partly redefined by a recent awareness of the role of race, ethnicity but also lifestyle and other social determinants. By contrast, the growth in rank and volume of another subdomain, evaluation of treatment regimens^L2D124^, goes back to earlier periods and can be related to the increased availability of new drugs and drug regimens since the early 2000, a trend that also accounts for the growth of the of treatment side‐effects^L2D131^ subdomain. Finally, the rise of the meta‐analysis^L2D186^ subdomain translates a growing preoccupation with regulating the increasing flow of biomedical information (Moreira, 2007).

Concerning subdomains that experienced a decrease in rank, two subdomains stand out. While we did observe that “quality” is a major concern for the oncopolicy domains, we also notice that its pain management^L2D151^ aspect drops to the very bottom of the rank hierarchy, and the quality of life and treatment side effect's^L2D120^ subdomain appears to be on its way out of the top ranks: in both cases it can be argued that, in contrast to other aspects of the quality of life bundle, these aspects are relatively well managed and part of routine clinical interventions rather than the topic of a translational research conference such as the ASCO meetings. A similar explanation could apply to the regular drop of the breast cancer screening^L2D134^ subdomain that refers to issues that have been around for some time, such as mammography screening and genetic testing for hereditary cancer, although additional qualitative investigations should be used to better understand this latter trend. Finally, a few domains, for instance “assessment of physical and psychosocial side effects”^L2D147^ and “survey questionnaires”^L2D153^, oscillate between ranks during the six periods, a fact probably due to annual contingencies rather than to underlying trends, although here too additional qualitative research spurred by these computational results would be needed to get a better understanding of the temporal dynamics.

Before we conclude our analysis, we very briefly address, without thoroughly presenting these results, a few additional procedures afforded by our approach. First, we took the oncopolicy domains and treated them as a self‐standing corpus, to which we fitted a new domain‐chained model of conference years to obtain a partition into periods that reflects only the evolution of these domains. This yields three single level periods (1995–2005, 2006–2012, 2013–2017). We then considered the later of these oncopolicy‐specific periods to represent its more current research trends and restricted once more the corpus, keeping only documents found in both oncopolicy and (2013–2017). To this last subcorpus, we fitted a domain‐chained model of the countries found in the affiliation metadata, thus clustering them from their insertion in “current oncopolicy.” Using a country‐domain table to investigate these clusters shows, for instance, that the bulk of European countries, together with Brazil, form a single cluster displaying a slight tendency toward “Meta‐analysis of clinical trials”^L2D186^ and “Quality of life and treatment side effects”^L2D120^. Analogous procedures could mobilize other domains and metadata, for example, to contrast research strategies of institutions and funders across and within domains.

## 5 DISCUSSION

We have shown how domain‐topic models and domain‐chained models, allied with measures, tables, and maps tailored to render model states associated with data accessible to human researchers, afford a fine‐grained and multifaceted view over the scientific dynamics of the ASCO Annual Meeting. The domain‐topic model formed the basis of our approach. By abstracting topic‐wise similar documents into domains, and domain‐wise similar terms into topics, at different levels of scale, it systematized and facilitated the navigation and characterization of the corpus.

Applying a domain‐chained model to the meeting's years allowed us to identify content based periods, whose consistent chronology reflects the conference's unfolding dynamics. Through these periods we located and broadly described a few shifting domains, including a growing level 3 domain related to “public health” and of interest to major current events in ASCO and other oncology organizations. We chose to focus on this issue, and by inspecting the topics of this domain we located and expanded our scope to another, strongly related level 3 domain. We investigated these two domains upward from their concrete elements, characterizing their direct subdomains, the domains themselves, and the ensemble we label “oncopolicy.” In sequence, by crossing periods and characterized subdomains, we provided an analytical picture of the evolution of “oncopolicy.” And, lastly, we have briefly exemplified how to employ sequences of operations with domain‐chained models and the production of targeted sub‐corpora in order to perform more complex inquiries into the data.

To further illustrate the heuristic value of our approach, we refer to a recent article (Pallari et al., 2021) that documents the differential impact on clinical practices of different kinds of research. It does so by investigating citations included in Clinical Practice Guidelines (CPGs) for lung cancer, that is, statements by professional or other regulatory bodies that feature recommendations intended to optimize patient care. Their results show that:

The types of research cited by the CPGs were primarily clinical trials, as well as three treatment modalities (chemotherapy, radiotherapy and surgery). Genetics, palliative care and quality of life were largely neglected.

While one would expect that articles on clinical trials and the three major treatment modalities will act as primary evidentiary sources for CPGs, it is somewhat surprising that this is not the case for contributions on topics such as “quality of life,” which, as we saw, are increasingly discussed during ASCO meetings. This raises a number of interesting questions, for instance, how to account for such a discrepancy between these two evidentiary settings? And, relatedly, what translational mechanisms are implemented to operationalize “matters of concern” (Callon & Rabeharisoa, 2008) such as quality of life and quality of care? Two fields of practice constitutive of population and global health that, as recently emphasized by ASCO's Chief Medical Officer (Goldberg, 2020), are increasingly part of the activities of professional clinical bodies. Our results offer a path into these inquiries, by mobilizing domains as analytic categories combining topics such as “chemotherapy” and “lung cancer” (as witnessed for L1D458), and “quality of life” and “clinical guidelines” (as seen with L2D153), together with nontextual dimensions that may inform the sociotechnical conditions for translational strategies.

In line with a recent special issue of Quantitative Science Studies (Leydesdorff et al., 2020) that called for a new alliance between computational and qualitative investigations of techno‐scientific activities, our goal has been to show one way in which advanced statistical techniques, deployed in a modular fashion, may provide rigorous yet flexible abstractions that enable original insights, which can be complemented by qualitative investigation and fed back into the inquiry, in a disciplined cycle. Moreover, and in the spirit of providing a vocabulary contributing to the establishment of such trading zones between computational and qualitative approaches, one can argue that domains, as defined in this paper, could be equated in more qualitative terms to epistemic communities (Akrich, 2010), whereby topics would amount to the discursive resources mobilized by those epistemic communities and acting as bridges between them.

This work also hints at a few questions to be addressed by further research, in respect to the approach we introduced and the corpus we studied. Concerning the approach, although we make a strong case for our choice of model, one may ask whether other models, such as those discussed in the introduction, could be expanded to perform a similar role. As a result, a quantitative or systematic method of comparison with such alternatives is desirable. Another interesting avenue would be to consider small corpora that do not generate a detailed topic partition on their own, yet may borrow a reference topic partition from a larger and thematically related corpus, which could afford the discovery of domains in the small corpus by employing the same procedure as the chained‐model presented here. Concerning the corpus, it would be interesting to compare the ASCO Annual Meeting with other conferences such as ESMO or the smaller ASCO meetings, by modeling them together and observing, for example, their differential insertion in domains. Beyond that, studying conferences, such as the ASCO Annual Meeting, together with related grant proposals and publications may provide an original view into research cycles if one can account for the diversity of these corpora and their metadata, a task for which our approach seems an able candidate. We conclude with a note, that the methods introduced, while built around the study of scientific activities, may be applicable to the study of other subjects, so long as the subject is associated with a corpus whose documents play a connective role across its dimensions.

## CONFLICT OF INTEREST

The authors declare no potential conflict of interest.

## AUTHOR CONTRIBUTIONS

**Alexandre Hannud Abdo**: Conceptualization, Data curation, Formal analysis, Investigation, Methodology, Resources, Software, Validation, Visualization, Writing—original draft, Writing—review & editing. **Jean‐Philippe Cointet**: Conceptualization, Funding acquisition, Methodology, Resources, Validation, Writing —original draft, Writing—review & editing. **Pascale Bourret**: Conceptualization, Methodology, Resources, Validation, Writing—original draft, Writing—review & editing. **Alberto Cambrosio**: Conceptualization, Funding acquisition, Methodology, Project administration, Resources, Supervision, Validation, Writing—original draft, Writing—review & editing.

## Data Availability

A software library to perform the procedures described in this paper is available at https://gitlab.com/solstag/abstractology/ as FLOSS (GPLv3). It depends on the graph‐tool library (Peixoto, 2014a). Most of the procedures described in this paper are also made available for noncoders through the CorText Manager on‐line data analysis service, see https://docs.cortext.net/sashimi/. The ASCO retains the rights to the raw data. Those interested in obtaining the full dataset for research purposes should contact ASCO. See https://www.asco.org/research-progress/asco-data-library. Postprocessed data is available as Appendix S1 (in SI‐DATA) that should suffice to use the inferred domain‐topic model to reproduce the other results and conduct further analysis at the same level of detail discussed in this paper. Readers must obtain the contents of abstracts from ASCO to infer the domain‐topic model themselves. [SI‐DATA, SI‐MAPS and SI‐TABLES available from https://doi.org/10.5281/zenodo.3596035].

## Appendix A THE ASCO ANNUAL MEETING ABSTRACTS

Science studies scholars have rarely investigated scientific conferences. Notable exceptions include Söderqvist and Silverstein (Söderqvist & Silverstein, 1994a, 1994b) who analyzed immunology meetings to unravel the sub‐disciplinary structure of that discipline. Their approach, however, was based on a cluster analysis of meeting participants, rather than an investigation of the content of presentations. A more recent study of scientific conferences has focused on a comparison of ASCO and its European counterpart, ESMO, between 2000 and 2010 (Pentheroudakis et al., 2012). This study was confined to the analysis of presentations related to clinical trials, and focused on differences between the two meetings in terms of a small number of a priori defined parameters such as industry sponsorship, blinded design, and sample size.

So why ASCO? The ASCO Annual Meeting is the main venue for cancer researchers from around the world to present innovative results. Established in 1964 with 66 members specializing in the then emerging chemotherapy domain, ASCO membership had grown by 2010 to more than 27,000 adherents representing all oncology subspecialties. Its network presently connects close to 45,000 oncology professionals and covers more than 150 countries. Attendance at the annual meeting broke the 5,000 mark in 1985, and in 2018 had increased to 40,700 participants. Regularly attended by a large number of foreign practitioners (46% of all attendees in 2018), the ASCO Annual Meeting has become one of the largest gatherings of medical professionals in the world. Initially held in conjunction with the annual meeting of the American Association for Cancer Research (AACR), the ASCO conference became self‐standing in 1993, and, according to its organizers, by 1995 a decision was made to increase its emphasis on translational science. We can thus safely claim that papers selected for presentation at ASCO provide a representative sample of oncology investigations at the international research front, often including practice‐changing results.

Figure 5 shows the evolution of the number of abstracts presented at the ASCO Annual Meeting from 1995 to 2017, reaching a plateau of approximately 5,000 abstracts in the 2010s. There are both practical and theoretical reasons to examine these abstracts and select this 23‐year window. Oncology has played a pioneering role in the much‐touted area of translational research (Cambrosio et al., 2006), a subfield characterized by rapid change. And as just mentioned, 1995 coincides with a readjustment of the socio‐technical content of the annual meetings that continues to characterize them to the present day. We can therefore test our approach on a reasonably stable, coherent set of documents that pertain, at the same time, to a dynamic domain. The highly selective nature of the ASCO Annual Meeting will moreover dispense us with the thorny issue of separating the grain of what practitioners consider innovative contributions from the chaff of the mundane or routine findings that can be found in a rapidly expanding number of oncology journals

Finally, we note that presentations at ASCO meetings are distributed throughout different conference sections and sessions. However, these “native” categories are primarily designed for organizational purposes, and thus highly contingent. By contrast, analyzing the abstracts' raw textual material provides a direct take on the thematic landscape of the conference as a whole and across time.

## Appendix B ASCO DOMAIN‐TOPIC TABLES

To produce domain labels, we resort to the domain‐topic table for domain L3D44. It is, however, impractical to include the full table here, as under the 10 level 2 domains it contains about a hundred level 1 domains. To illustrate the procedure, we will show an extract of the table for one level 2 domain, and then a resulting table containing all 10 level 2 labels but omitting the level 1 details required to formulate them. The full domain‐topic tables are available to readers as Appendix S1 in SI‐TABLES.

Table B1 presents the row for domain L2D120 from the aforementioned domain‐topic table. As explained in Section 2.3, individual level 1 domains can be more directly interpreted since they are substantively coherent, with their assemblage of topics featured in the documents they contain. Additionally, one may as needed resort to other details such as document titles or contents. By inspecting the table and interpreting the ensemble of its level 1 subdomains, we arrive at an appropriate label for L2D120: “quality of life and treatment side‐effects”.

Going through this process for each of the 10 subdomains of L3D44 yields the labels presented in Table B2, which also provides the size and a summary for each level 2 domain. Then, by looking at the ensemble of these level 2 labels, we can provide a label for the entire level 3 domain: “public health and health technology assessment (broadly understood to include treatment regimens),” which we associate with the native yet vague term “oncopolicy.”

Still, one may ask if we are not losing some meaningful related material by focusing only on this single level 3 domain. To answer that, we look at the topics common to its subdomains, and notice the main shared topic is L1T127, whose most shared term is “health_care”. Going back to the domain‐topic map and selecting this topic, it becomes clear that our chosen domain dominates the usage of topic L1T127 (see Figure 4). Yet, a single other level 3 domain still stands out: L3D48. By similarly inspecting the next common topics for the first domain, we can see that this other domain, despite their differences, also fits within the same realm of oncopolicy. We therefore proceed to include it in our analysis. The resulting labels and summary information are presented in Table B3, evidencing the domain's specific focus on cancer screening and risk factors.

**TABLE B1:** Line L2D120 from the domain‐topic table for L3D44

L2	Common topics	L1	Specific topics
D120	• T156 (9%): **repeated_measures** (100%) • T67 (8%): **questionnaire** (76%) • T14 (8%): **scores**(28%), score (20%), scale (16%) • T272 (7%): **baseline** (45%) • T23 (6%): **pain** (35%), symptoms (34%), severity (20%) • T241 (4%): **randomized** (50%) • T12 (4%) • T249 (3%): **qualit**y (54%), life (40%) • T367 (3%): **measured** (38%), effect (33%), effects (24%)	D415	T156 (23%): **qol** (3%), scales (3%), life_qol (2%), subscales (2%), internal_consistency (2%) T67 (15%): **items** (6%), item (3%), questionnaire (3%) T14 (10%): **scale** (12%), scores (9%), validity (9%), reliability (8%), measure (8%) T23 (4%): **symptoms** (17%), symptom (14%), pain (13%)
		D563	T67 (42%): **exercise** (2%), depression (2%), physical_activity (2%), physical (1%), anxiety (1%) T249 (5%): **intervention** (32%) T156 (4%): **qol** (13%), life_qol (8%)
		D584	T156 (30%): **qol** (6%), eortc_qlq‐c30 (4%) T14 (6%): **scores** (24%), score (16%) T67 (5%): **questionnaire** (6%), physical (6%), questionnaires (5%), items (3%) T241 (4%): **arms** (19%), arm (17%), randomized (17%) T272 (4%): **baseline** (41%) T23 (3%): **symptoms** (17%), pain (11%), deterioration (10%)
		D614	T134 (54%): **bone** (4%), bone_metastases (4%), zoledronic_acid (3%), bone_resorption (2%)
		D635	T38 (54%): **weight** (3%), bmi (3%), body_composition (2%), cachexia (2%), nutritional_status (2%)
		D687	T12 (28%): **cipn** (3%), chemotherapy‐induced_peripheral (2%), neuropathy_cipn (2%), hands (2%), feet (1%) T23 (4%): **severity** (18%), pain (16%) T79 (4%): **neuropathy** (13%), peripheral_neuropathy (9%), neurotoxicity (7%) T142 (3%): **placebo** (30%) T181 (3%): **skin** (14%), topical (10%), cutaneous (9%) T241 (3%): **randomized** (21%), arm (16%), arms (15%) T28 (2%): **daily** (22%) T283 (2%): **side_effects** (20%), severe (17%), side_effect (17%) T355 (2%): **incidence** (24%), prevention (24%)
		D805	T40 (19%): **approval** (4%), fda (4%) T228 (5%): **drug** (38%) T93 (3%): **approved** (15%) T65 (3%): **ae** (9%), aes (8%), sae (5%), saes (5%) T251 (3%): **design** (26%) T236 (3%): **review** (14%), reports (13%), reporting (10%), published (7%) T292 (2%): **trials** (59%) T29 (2%): **maximum_tolerated** (14%), mtd (12%), dlt (9%), dose_escalation (9%) T11 (2%): **supplemental_indications** (2%), marketing (2%), notification (1%), biopharmaceutics (1%), budgeted (1%) T395 (2%): **phase** (81%) T258 (2%): **clinical_trials** (72%) T240 (2%): **Safety** (29%) T239 (2%): **new** (35%) T230 (1%): **accrual** (32%)

*Note*: Percentages are the item's fraction of total positive similarity (or commonality) contributions, either at the topic or term level. Topics are always level 1. Note that L1T12 has no terms present in all subdomains of L2D120.

**TABLE B2:** Complete description of the “public health and health technology assessment”^L3D44^ domain in terms of its common topics and subdomains

**L3D44**			
**Label:** Public health and health technology assessment (broadly understood to include treatment regimens)			
**Common topics:** T127: **Health_care**, physicians, physician, community · T343: **Care**, medical, health · T58: **Utilization**, claims · T135: **More_likely**, adjusted, demographic, logistic_regression · T33: **Less_likely**, receipt · T260: **Use** · T72: **Hospital**, national, center · T102: **Should_be**, regarding, they, should · T222: **Practice**, guidelines · T325: **Over**, period · T96: **Issues**, how, clinic, needs, participants · T15: **Age**, older, mean_age · T249: **Quality**, life · T84: **Cost**, costs · T304: **Outcomes**, impact · T150: **Comorbidities**, comorbidity · T198: **Among**, those · T116: **Hospitalization**, outpatient, palliative_care, inpatient, length · T187: **More**, most · T14: **Score**, measures, scores · T273: **Population**, us, proportion · T341: **Diagnosis**, diagnosed · T215: **Clinicians**, actual, preferred, decision, preference · T170: **Compared**, than, lower, greater, higher			
**L2 subdomains**			
**L2**	**Label**	**N**	**Summary**
D120	*Quality of life and treatment side effects*	1,796	Quality of life (physical and psychological). Treatment side effects.
D121	*Quality improvement*	2,303	Quality improvement (professionals, costs, and practices). Professional education and communication with patients.
D124	*Evaluation of treatment regimens*	1,343	Comparative evaluation of treatment regimens (resources, costs, efficacy, side effects).
D126	*Epidemiological surveillance of outcomes*	1,931	Epidemiological surveillance of outcomes (SEER = surveillance, epidemiology, and end results). Prognosis.
D131	*Risks and management of treatment side effects*	1,387	Risks and management of treatment side effects (toxicity, infection, etc.)
D147	*Assessment of physical and psychosocial side effects*	1,523	Assessment of physical and psychosocial side effects. Hospitalization.
D151	*Pain management and quality of life*	805	Pain management and quality of life (randomized studies thereof).
D153	*Survey questionnaires of quality of life and professional adherence to guidelines*	1,844	Survey questionnaires of quality of life (including depression and anxiety). Survey of professional adherence to clinical guidelines.
D163	*Patient lifestyle education*	1,142	Patient education concerning lifestyles. Barriers to screening.
D186	*Meta‐analysis of clinical trials*	953	Meta‐analysis of clinical trials (all sorts of cancers).

*Note*: Level 2 subdomains were labeled by employing the domain‐topic table for the level 3 domain, whose row describing the “quality of life and treatment side effects”^L2D120^ subdomain was presented as Table B1.

**TABLE B3:** Complete description of the “screening and risk factors for cancers”^L3D48^ domain

**L3D48**			
**Label:** Screening and risk factors for cancers (in particular hereditary and secondary cancers), including epidemiological surveillance			
**Common topics:** T80: **Screening** · T97: **Family_history** · T73: **Seer**, surveillance_epidemiology, white, seer_database, race · T33: **Population‐based**, registries · T341: **Diagnosis**, diagnosed, registry · T109: **Mortality**, general_population · T3: **Country**, countries, world · T72: **National**, center · T96: **Participants** · T127: **Health_care**, community, physicians, referral, physician · T78: **Survey**, education · T15: **Age** · T246: **Risk** · T47: **Invasive**, mammography · T273: **Population** · T222: **Guidelines**, recommendations · T242: **Cancer**, cancers · T362: **Breast_cancer**, breast			
**L2 subdomains**			
**L2**	**Label**	**N**	**Summary**
D134	*Breast cancer screening and prophylaxis*	588	Screening (mammography and genetic testing) for breast cancer, esp. hereditary one, and prophylaxis, including practice guidelines
D139	*Genetic testing and counseling for hereditary cancer risks*	509	Genetic testing and genetic counseling for hereditary cancer risks. Epidemiological surveillance
D141	*Cancer risk factors*	433	Cancer risk factors, including race, ethnicity, smoking, and obesity
D159	*Epidemiology of cancer*, *esp*. *blood cancers*	426	Epidemiology of cancer, esp. blood cancers. Risk factors for secondary cancer (following treatment of primary cancer). Epidemiological surveillance in different world populations
D180	*Molecular pathology: Quality control*, *technology comparisons*, *and regulation*	342	Molecular pathology: Quality and comparison of different technologies. Molecular testing and its regulation, access to testing, expertise, and decision support for testing (both human expertise and computational approaches)
